# Nondestructive Mechanical and Electrical Characterization of Piezoelectric Zinc Oxide Nanowires for Energy Harvesting

**DOI:** 10.3390/mi16080927

**Published:** 2025-08-12

**Authors:** Frank Eric Boye Anang, Markys Cain, Min Xu, Zhi Li, Uwe Brand, Darshit Jangid, Sebastian Seibert, Chris Schwalb, Erwin Peiner

**Affiliations:** 1 Instute of Semiconductor Technology, Technische Universität Braunschweig, 38106 Braunschweig, Germany; 2 Scientific Metrology Department, Ghana Standards Authority (GSA), Accra P.O. Box MB 245, Ghana; 3 Electrosciences Ltd., Farnham GU9 9QT, UK; markys.cain@electrosciences.co.uk; 4 Surface Metrology Department, Physikalisch-Technische Bundesanstalt (PTB), 38116 Braunschweig, Germany; min.xu@ptb.de (M.X.); zhi.li@ptb.de (Z.L.); uwe.brand@ptb.de (U.B.); 5 Quantum Design Microscopy (QDM) GmbH, 64319 Pfungstadt, Germany; jangid@qd-microscopy.com (D.J.); seibert@qd-microscopy.com (S.S.); schwalb@qd-microscopy.com (C.S.); 6 Laboratory for Emerging Nanometrology (LENA), Technische Universität Braunschweig, 38106 Braunschweig, Germany

**Keywords:** piezoelectric nanogenerator, zinc oxide nanowire, SEM, contact resonance, piezoelectric coefficient *d*_33_, electrical probing

## Abstract

In this study we report on the structural, mechanical, and electrical characterization of different structures of vertically aligned zinc oxide (ZnO) nanowires (NWs) synthesized using hydrothermal methods. By optimizing the growth conditions, scanning electron microscopy (SEM) micrographs show that the ZnO NWs could reach an astounding 51.9 ± 0.82 µm in length, 0.7 ± 0.08 µm in diameter, and 3.3 ± 2.1 µm^−2^ density of the number of NWs per area within 24 h of growth time, compared with a reported value of ~26.8 µm in length for the same period. The indentation modulus of the as-grown ZnO NWs was determined using contact resonance (CR) measurements using atomic force microscopy (AFM). An indentation modulus of 122.2 ± 2.3 GPa for the NW array sample with an average diameter of ~690 nm was found to be close to the reference bulk ZnO value of 125 GPa. Furthermore, the measurement of the piezoelectric coefficient (*d*_33_) using the traceable ESPY33 tool under cyclic compressive stress gave a value of 1.6 ± 0.4 pC/N at 0.02 N with ZnO NWs of 100 ± 10 nm and 2.69 ± 0.05 µm in diameter and length, respectively, which were embedded in an S1818 polymer. Current–voltage (*I-V*) measurements of the ZnO NWs fabricated on an *n*-type silicon (Si) substrate utilizing a micromanipulator integrated with a tungsten (W) probe exhibits Ohmic behavior, revealing an important phenomenon which can be attributed to the generated electric field by the tungsten probe, dielectric residue, or conductive material.

## 1. Introduction

Since the introduction of the first piezoelectric nanogenerator (PENG) in 2006, many efforts have been made by researchers to advance the technology into large-scale applications. This stems from the low output of ~6.5 mV realized from the device when an atomic force microscope (AFM) with a platinum (Pt) tip was applied to an array of zinc oxide (ZnO) nanowires (NWs) to deform them [1]. The main challenge, which has been of great interest, is how to enhance the output performance of such a novel device. Many research findings have suggested that the output performance improvement of PENGs largely depends on the nanowires’ geometry (length and diameter). As can be expected, the piezoelectric coefficient of ZnO NWs, for instance, increases with an increase in NW height and a decrease in their diameter, thereby resulting in a higher output potential [2]. Longer and thinner nanowires with higher aspect ratios have been investigated to assess the potential of improving the overall output voltage [3]. This has been demonstrated in the simulation results of Oliveira et al. [2]. However, few experimental investigations have been conducted and reported to establish this assertion.

The NW lengths in this work, using an improved thermo-convective solution growth (TSG) method [4], are shown to be considerably increased compared with our conventional chemical bath deposition (CBD) growth method [5,6,7]. In the work of Ou et al., where the hydrothermal synthesis method was used, the ZnO NW length and diameter could reach ~12 µm and ~250 nm, respectively, only after refreshing the precursor solution twice [8]. This approach, which was preceded by the dip coating of a ZnO seed layer, has the disadvantage of limited mass transport growth and the wasteful use of chemicals.

In our previous work, the influence of the seed layer (SL) thickness on the morphology of nanowires was investigated [4]. An optimal SL thickness of ~10 nm of a Zn thin film on a silicon substrate was achieved after 10 min of direct current (DC) sputtering at room temperature, and subsequent annealing to form a ZnO SL. The ZnO SL served to minimize the thermodynamic barrier for ZnO crystallization by providing nucleation sites and hence facilitating the growth of vertically aligned ZnO NWs [9]. The SL equally has the characteristic of enhancing the NW arrays’ alignment and density [9], compared with the seedless approach [10]. Furthermore, an advantage of this method over the direct deposition of ZnO is the optional patterning for integration, e.g., into junction piezotronic transistor arrays [11] or on microelectromechanical systems (MEMS) cantilevers [5,6] using selective area growth (SAG) on a patterned SL fabricated by the lift-off photolithography of Zn deposited by room temperature sputtering. A resultant nanowire length of ~27 µm, aspect ratio of ~50, and density of 1.9 µm^−2^ was realized using TSG [12]. However, we noticed that there was a tendency towards the inhomogeneous deposition of the precursor solution and, therefore, non-uniform growth on the substrate surface. This was because there was no form of agitation of the reaction solution, leading to severe precursor precipitation at the bottom of the reaction container. During the further optimization of the TSG method, we therefore introduced magnetic stirring (at a speed of 250 rpm) and an air pump (flow rate of 300 mL/min) into the precursor solution during the growth process. These additional techniques were aimed at ensuring continuous mixing and a more homogeneous distribution of the precursor solution onto the silicon (Si) substrate surface [13].

On the choice of materials, ZnO was selected because of its large bandgap (3.4 eV), semiconducting properties, and piezoelectric properties among semiconductors. It has the largest piezoelectric response of approximately 2 to 12 pC/N [14,15,16,17], compared with any tetrahedrally bonded semiconductor, allowing for strong piezoelectric polarization. Moreover, it is a biocompatible material [12,18]. Compared with other semiconductor materials such as gallium nitride (GaN) and aluminum nitride (AlN), ZnO is a readily available material and can easily be grown using a variety of methods [18], on different substrates, and at low temperatures (<105 °C) [19]. ZnO NWs have emerged as a viable building block in recent years for a variety of micro and nano devices used in energy harvesting [20,21], photonics, sensors [5,22], sensing devices, e.g., in the context of healthcare [23,24], actuators, solar cells, and many other applications [25].

Considering the wide range of applications of ZnO NWs in energy harvesting and sensing, the characterization of their structural, mechanical, and electrical properties is of great importance. Typical structural characterization techniques include SEM, which provides insights into the morphology and alignment of the NWs. The high-temperature thermal treatment (calcination) of NWs plays a vital role in tuning the structure, defect levels, and ultimately their piezoelectric performance. Calcination in an O_2_ atmosphere in a typical temperature range of (700–900) °C reduces carbon contamination, improves crystallinity, and thereby adjusts oxygen vacancy and electron densities, which control the screening of piezoelectric response. The dual effect of increased surface oxygen vacancy concentration but reduced trap densities enhanced the effective piezoelectric coefficient (*d*_33_^eff^) by ~47%, as reported by Legardinier et al. [26]. In addition, Raman spectroscopy characterization offers insights into crystal quality (phase quality of wurtzite-structured ZnO), defect levels, and the impact of stress, all of which affect piezoelectric performance. X-ray diffraction (XRD) is used to confirm the crystalline structure, often revealing a wurtzite phase with preferential growth along the *c*-axis. These investigations have been carried out in detail and reported in our previous publications [4,7]. However, NW calcination for the further improvement of *d*_33_ has neither been considered there, nor in the current study, which focuses on optimizing the growth enhancement of ZnO NWs using TSG. Due to its potential, we will investigate in our next study the effect of calcination on the mechanical, electrical, and piezoelectric properties of ZnO NWs fabricated using TSG, e.g., by high-temperature treatment in the range of (600–900) °C [26].

The characterization of the mechanical properties of ZnO NWs gives information about their internal structure, which helps as a guide for performance improvement and application [27,28]. Furthermore, in piezoelectric energy harvesting, the Young’s modulus and fracture strength of the NWs need to be investigated to determine the maximum force that can be applied without device failure. Correspondingly, the investigation of mechanical properties [28,29] of ZnO NWs for sensing and energy harvesting purposes is an important factor for their efficient application. This is because such nanomaterials, used for nano devices, often undergo considerable mechanical deformation. Their mechanical and long-term stability in performance is therefore of paramount importance, which depends on the NW size [28], to a large extent. For maximum output in piezoelectric nanogenerators, for instance, the device is expected to be operated in compressive mode, i.e., using vertically aligned NWAs in a vertically integrated nanogenerator (VING). It is therefore important to have an accurate and comprehensive understanding of the mechanical properties of such nanostructures for their appropriate integration in a nano device. More specifically, in a VING composed of piezoelectric NWs embedded in a stabilizing matrix, the NWs should have a larger Young’s modulus than the embedding material, such that the strain in the NWs exerted by an axial compressive force is retained, whilst the stiffness of the top layer has a much lower effect [24].

The investigation of the electrical properties is intended to provide an understanding of the role of the metal–semiconductor interface and possible short-circuit effects by the embedding polymer in VINGs. For electrical characterization, it is important to note that the piezoelectric properties of ZnO NWs have often been characterized using a two-point probe approach for a single NW. In this process, two tungsten probes are used, in which one is used to hold the NW in position, while the other deforms it. A digital multimeter is then used to measure the generated voltage potential resulting from NW deformation [27]. This method may not be transferable to VINGs, since for efficient and optimum energy harvesting, an array of NWs is required, and these wires may not have the same dimensions. Therefore, piezoelectric output performance characterization must be performed on many single NWs to provide a statistically relevant parameter related to the performance of the VING. This has to be considered as well for measuring the conductivity of ZnO NWAs using a micromanipulator-coupled tungsten probe [30,31,32]. By analyzing the current–voltage (*I-V*) characteristics of ZnO NWAs, information regarding Schottky or Ohmic behavior of the electrical contacts of the nanogenerator device is provided. As can be expected in piezoelectric energy harvesting, the semiconductor–metal interface (ZnO NWs/Au) at the top of the device should show a rectifier behavior, whereas the bottom contact between ZnO NW and Si substrate is Ohmic [7]. In that case, the flow of current generated by the device is controlled in a particular direction.

Despite the relevance of this information for the performance improvement and optimization of nanoscale devices, which have gained prominence, little experimental research in this area has been reported. Bestley et al. [33] investigated the displacement, acceleration, and pressure characteristics of ZnO nanowires employing numerical modeling. In their findings, a total displacement of 4.6 nm along the nanowire (*L* = 3 µm; *D* = 312.5 nm) was observed at an eigen frequency of ~572 Hz. The simulations also showed that the NW can deform beyond a pressure of 575 Pa. However, there was no experimental data to support this assertion. Mention can also be made of Briscoe et al., who evaluated the capacitance–voltage (C-V), current–voltage (*I-V*), and impedance spectroscopy characteristics measured with their device through equivalent circuit modeling [34]. Their results were, however, limited to information about the capacitance, internal resistance, and rectification behavior of the NG device.

Therefore, in this study, we delve into the characterization of ZnO NWs with an emphasis on their synthesis using an optimized TSG method, regarding mechanical, electrical, and piezoelectric properties, and the implications for energy harvesting. The indentation modulus of our fabricated ZnO NWs was characterized using an AFM in contact mode. For the electrical characterization of single NWs, we employ micromanipulators as an add-on to the Quantum Design FusionScope. The piezoelectric properties of ZnO NWAs with top and bottom electrodes are investigated using an Electrosciences ESPY33 measurement tool, which is suitable for the traceable characterization of piezoelectric materials, such as piezoelectric ceramics, single crystals, piezoelectric polymers (e.g., Polyvinylidene Fluoride, PVDF), and composites [35]. It measures the charge developed within a piezoelectric material that undergoes a cyclic compressive force application [7,36].

The findings in this study are crucial for understanding the mechanical and electrical properties of ZnO NW-based piezoelectric devices and are also important for estimating the real power conversion efficiency of PENGs [30].

## 2. Materials and Methods

### 2.1. Fabrication of ZnO Nanowires

In this fabrication process, an *n*-type, phosphorus-doped Si wafer with <100> crystal orientation purchased from Siegert Wafer GmbH, Aachen, Germany, was used. The Si wafer has a thickness of 525 ± 20 µm, a diameter of 100 ± 0.3 µm, and a resistivity of 1–5 Ω × cm. For basic cleaning to remove the native oxide layer, the Si substrate was cut into the required sizes and dipped in a hydrofluoric (HF) acid mixture consisting of 30% H_2_O_2_ and 96% H_2_SO_4_ in a volume ratio of 1:1 for 5 min. After the HF dip, the samples were rinsed in deionized (DI) water to remove the residual HF solution and finally dried in a stream of nitrogen (N_2_) gas.

Before ZnO NW growth, phosphorus doping on the back and front sides of the Si substrate was performed using the spin-on dopant P509, supplied by Filmtronics Inc., Butler, PA, USA. Spin coating was performed at a speed of 3000 rpm for 30 s, with subsequent diffusion in a diffusion furnace at 1100 °C for 30 min in an O_2_/N_2_ ambient of (0.5/4) L/min volume (or mass) fraction, respectively. Phosphorus doping of the Si substrate serves to increase conductivity and facilitates efficient charge collection from the bottom [7,37]. Finally, a Cr/Au bottom electrode of 50/500 nm thickness was deposited at the back of the Si substrate using electron beam (e-beam) evaporation (supplied by Leybold, Cologne, Germany).

The ZnO NWs were fabricated using two different NW growth methods. A first set of samples was fabricated using our conventional CBD growth process [5,7]. The ZnO NWs were grown on an *n*-type silicon wafer pre-deposited with a Zn thin film using DC sputtering and subsequent annealing (600 °C for 60 min) in ambient air conditions to form crystalline ZnO. In the standard CBD NW growth process, the precursor solution temperature was set at 90 °C, and a growth time of 3 h was used. These parameter settings (temperature and time) were optimized, and they enhance uniform and well aligned NW growth. Additionally, it is a low-cost approach, uses a low temperature (below the boiling point of water), and can be adapted for the selective area growth of NWAs for improved piezoelectric output [7]. After the growth of the NWs, AZ5214 E, S1818, and PMMA photoresists were used to encapsulate four sets each of the NW samples to investigate the effect of different polymer matrices on the output of the device. Despite AZ5214 E and S1818 being standard photoresists that are readily available and widely used in semiconductor technology, their mechanical properties, including tensile strength, which is influenced by processing conditions such as baking temperature and duration, make them potential candidates for nanowire encapsulation. They are easy to process (spin coat and develop) and have good mechanical cycling resistance [38,39,40]. These attributes necessitated their inclusion in this study, instead of the conventional polymers like PMMA and PDMS. However, further investigations towards their use as encapsulation polymers will be required for their compatibility with large-scale applications.

The AZ5214 E positive tone photoresist was spin coated on top of the NWs at a speed of 2000 rpm for 30 s, while the S1818 and PMMA were spin coated at 3000 rpm for 30 s to form encapsulating matrices. To ensure both polymers penetrated the NWs, the deposited polymers were allowed for 2 min (in the case of A5214 E and S1818), and 10 min (in the case of PMMA) to seep down into the NW spaces before spinning. The encapsulated NWs were then soft baked on a hot plate at 110 °C for 90 s for AZ5214 E, at 90 °C for 90 s for S1818, and at 110 °C for 90 s in the case of PMMA polymer. Before creating a top metal contact on the NWs, the polymer matrices were etched with O_2_ plasma (supplied by SENTECH Instruments GmbH, Berlin, Germany) to uncover the top part of the NWs (NW tips). The O_2_ plasma etch of the AZ5214 E polymer was performed for 5.5 min to have ~225 nm of the NW tips exposed, and for 7 min, resulting in ~270 nm of the NW tips being exposed in the case of S1818. For PMMA polymer etching, a time duration of 3 min gave ~200 nm of denuded NW tips. A 50/500 nm thick Cr/Au top electrode was then e-beam evaporated (supplied by Leybold, Cologne, Germany) on top of the NWs for all samples.

In the second set of samples, the ZnO NW arrays were grown on *n*-type silicon using our newly developed and optimized TSG nanowire growth method. In this method, the growth temperature is higher than in standard CBD and localized at the silicon substrate, which is attached to a holder mounted with a heat sink, thereby preventing the whole volume of solution from being heated. The advantages of this growth method are that it is a simple two-step process, avoids the waste of precursor solution, and avoids spurious deposition on the top of the nanowire arrays. It has been described in detail previously [4]. As in the case of the first set of samples, 50/500 nm of Cr/Au bottom metallization was first deposited on the back of the Si substrate. To improve the homogeneous growth of ZnO NWs on the top surface (covered with a ZnO SL), we introduced magnetic stirring of the precursor solution at a speed of 250 rpm in our TGS setup. The TSG results with magnetic stirring show uniformly grown and well aligned ZnO NW arrays compared with those without magnetic stirring. Simultaneously, by introducing solution stirring, the dimensions of the NWs became smaller compared with the TSG method without stirring, with a NW length reduction of about 52%. Given that, we further introduced an air pump (NICREW Aquarium air pump, purchased online from Amazon Services Europe S.à.r.l., L-1855 Luxembourg, Luxembourg, www.amazon.de (accessed on 25 March 2024)) into the solution. The air pump was operated at a flow rate of 300 mL/min to remove bubbles from the NWs’ surface that are combined with non-homogeneous growth and deposited particles after the growth process [13]. The air bubbles in the precursor solution facilitate the continuous mixing and homogeneous distribution of precursor solution above the sample surface, thereby avoiding the precipitation of precursors at the bottom of the growth container [13]. This enhances uniform and higher growth rates. Using this approach, we saw an increase of ~48% in NW lengths for TSG in the still precursor solution (without stirring). The fabricated ZnO NWAs were then encapsulated in S1818 and PMMA polymers by spin coating, as in the first set of samples. After the polymer matrix deposition, inductive coupled plasma (ICP) oxygen (O_2_) plasma (supplied by SUNTECH, Eschborn, Germany) etching was performed to expose the NW tips for subsequent top Cr/Au (50/500 nm thick) contact formation using e-beam evaporation. The S1818 and PMMA insulating matrices covering the NWs act to decrease the influence of external screening [7,41,42], enhance mechanical stability, and thereby increase the output potential signal strength.

**Figure 1:**
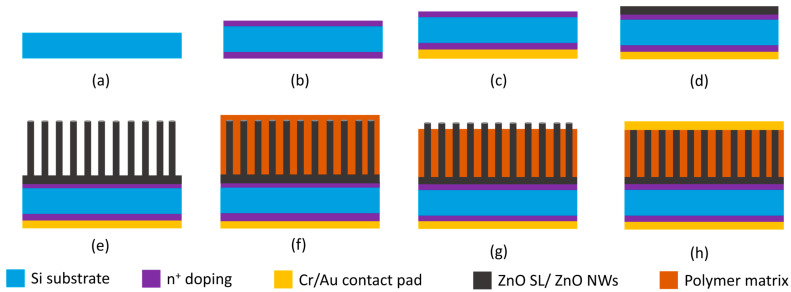
Fabrication steps of ZnO NW arrays on *n*-type Si substrate: (**a**) Si substrate; (**b**) *n*^+^ doping of Si for top and bottom contacts; (**c**) Cr/Au contact pad deposited on back of Si substrate using e-beam evaporation; (**d**) ZnO SL deposited using DC sputtering and annealing; (**e**) ZnO NW growth using CBD and TSG; (**f**) NW encapsulation in polymer matrix using spin coating; (**g**) O_2_ plasma etch of polymer matrix for top electrode deposition; (**h**) Cr/Au top electrode contact pad deposited using e-beam evaporation.

Inasmuch as the TSG method is an optimization of the CBD, their resultant NW dimensions cannot be directly compared due to the different setups (especially the heating mechanisms) and growth conditions employed. Firstly, in the CBD method, the entire volume of solution is heated at 90 °C, and therefore, to increase it to 105 °C, one can expect that the solution will begin to boil. This will cause increased evaporation and depletion of the precursor solution within a short period. Secondly, to increase the growth time to 24 h will mean that the precursor solution must be replaced at specific time periods (every 3–4 h) due to evaporation and depletion. This has the disadvantage of the wasteful use of chemicals and higher costs. Figure 1 shows a schematic of the step-by-step fabrication.

### 2.2. Characterization of ZnO Nanowires

#### 2.2.1. Contact Resonance (CR) Measurement

**Figure 2:**
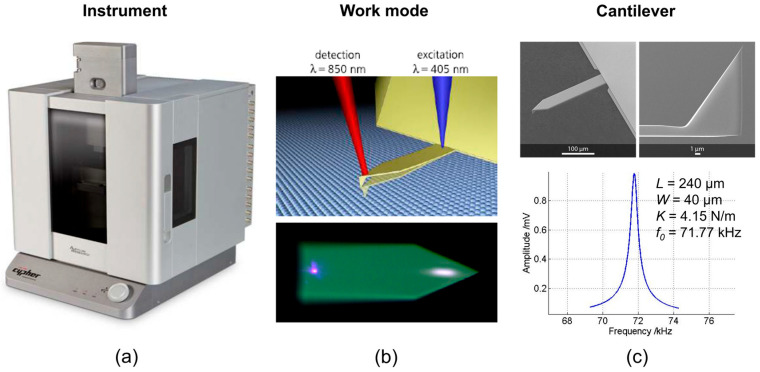
CR-AFM tool for measuring ZnO NWs arranged in a vertical array: (**a**) Asylum Cypher AFM; (**b**) schematic, upper, and optical micrographs, lower working mode of the cantilever at wavelengths of 850 nm for detection and 405 nm for excitation of resonant out-of-plane bending; (**c**) topand cross-sectional view SEM graphs, upper, and spectral shape of fundamental resonant bending mode, lower dimensions of cantilever used in the measurement with *L* = 240 µm and *W* = 40 µm, force constant and resonance frequency of 4.15 N/m and 71.77 kHz, respectively.

Fabricated ZnO NWs with diameters of ~100 nm, ~570 nm, and ~690 nm, denoted by CBD100, TSG570, and TSG690, respectively, were investigated for their elastic modulus in this study. The methods described in the literature for determining the elastic modulus of NWs comprise the MEMS-based tensile test, tensile/buckling test via a nanomanipulator, three-point bending, cantilever bending, resonance, or nanoindentation [43]. Mostly, these methods are performed with NWs suspended from their original substrate and assembled on a foreign holder or tool, and require a solid connection of the NWs there, e.g., using an adhesive. Only in the case of flexural bending and resonance through a nanomanipulator or an AFM can the NWs remain on their original substrate. Nevertheless, all these methods are designed to characterize separate NWs and are thus time-consuming and not suitable for high-throughput metrology with closely arranged vertical NW arrays. Different from these studies, nanoindentation combined with contact resonance AFM (CR-AFM) spectroscopy under vertical conditions, i.e., axial force application, was performed with silicon and GaN micropillar arrays [41,42]. Furthermore, axial CR measurements with NW arrays (silicon, copper, ZnO) were reported using a homebuilt piezoresistive microprobe imaging setup [24]. In the present study, we use the CR mode of an AFM integrated with an OPUS 240AC-NG cantilever with a length, *L* = 240 µm, width, *W* = 40 µm, and thickness, *b* = 2.6 µm. The AFM cantilever was used at a constant force of 4.15 N/m and a resonance frequency of 71.77 kHz. The BlueDrive Dual AC Tracking (DART) working mode, with detection and excitation wavelengths, *λ*_det_ = 850 nm and *λ*_exc_ = 405 nm, respectively, was used. The CR-AFM setup is shown in Figure 2.

**Figure 3:**
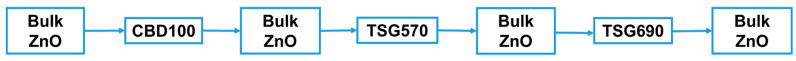
Procedure for the CR measurement of ZnO NWAs and a bulk ZnO crystal substrate using an AFM. CBD200 was measured separately.

The elastic modulus of our fabricated ZnO NWs was compared to that of a reference bulk ZnO crystal substrate purchased from MSE supplies, Tucson, AZ, USA. The reference ZnO crystal substrate had dimensions of 5 mm × 5 mm × 0.5 mm, a *c*-plane [0001] orientation, 99.99% purity, a hardness of 4 Mohs, and a surface roughness *Ra* < 0.5 nm. Due to the importance of tip radius and geometry in CR measurements, the following procedure in Figure 3 was followed: the measurement of the reference bulk ZnO crystal substrate was conducted before and after measuring the nanowires.

#### 2.2.2. Electrical Characterization

**Figure 4:**
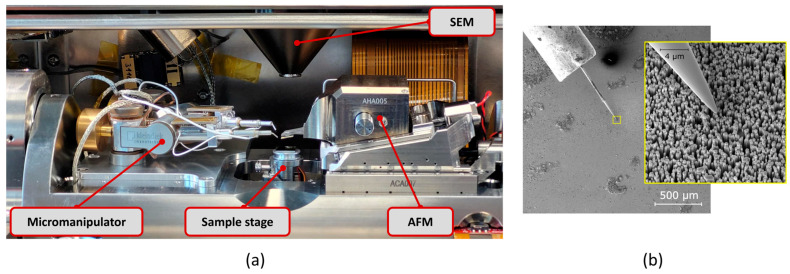
(**a**) Setup for measuring *I-V* characteristics of ZnO NWAs using a micromanipulator. Up to four microprobes can be integrated into the FusionScope simultaneously. (**b**) SEM micrograph at 25° tilted view of a tungsten probe in contact with a ZnO NW (TSG570).

The electrical characterization of the ZnO NWs was performed using micromanipulators. The electrical measurements were carried out in contact mode, with a conductive AFM tip in contact with the top of ZnO nanowires. To characterize the ZnO nanowires individually, *I-V* curves were acquired at three spots for each sample using tungsten probe tips (tip radius 100–150 nm) mounted on an LCMK (low-current measurement kit) attached to an MM3E micromanipulator (Kleindiek Nanotechnik, Reutlingen, Germany). The micromanipulators were mounted as an add-on to a FusionScope (Quantum Design, San Diego, CA, USA), which has integrated an AFM (atomic force microscope) and an SEM (scanning electron microscope) in one instrument [31]. The SEM imaging in tilt view up to 80° facilitates landing the probe tip precisely on distinct ZnO nanowires. The *I-V* curves were then acquired and processed using the digital EBIC (electron beam-induced current) amplifier and APT (Advanced Probing Tool) v4.338 software from Kleindiek Nanotechnik. The data from the *I-V* curve was processed using the SciDAVis software (version 2.7). The probing setup and an SEM micrograph of the probe making contact with a single NW are depicted in Figure 4.

The behavior of the *I-V* curve (Schottky or Ohmic) in the ZnO NWs after measurement largely involves the careful consideration of contact materials, fabrication techniques, and post-processing treatments. Understanding and controlling these factors are essential for the development of nanoscale electronic devices.

#### 2.2.3. Piezoelectric Coefficient d_33_

**Figure 5:**
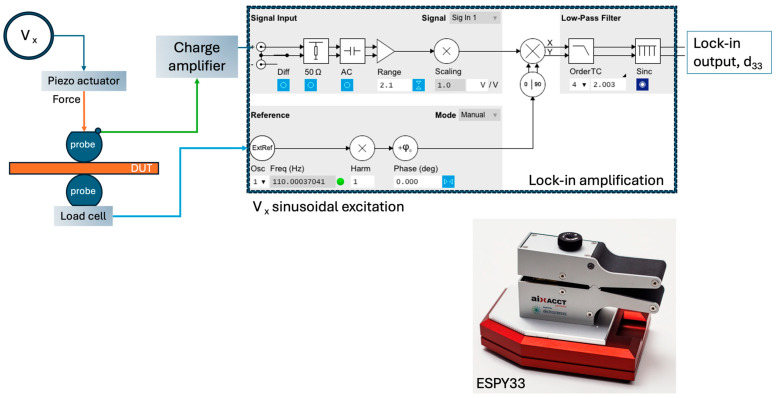
Schematic of ESPY33 measurement tool, its lock-in amplification circuit, and a photograph of the device.

The ZnO NWAs measured in this experiment have a length of ~2.7 µm, a diameter of ~100 nm, and an aspect ratio of 27 (CBD100, see Table 1). The Cr/Au top and back electrodes were connected to an ESPY33 piezoelectric measurement tool for measurement. The accurate and traceable measurement of the charge developed across the ZnO NWAs under cyclic compressive stress was performed using the ESPY33 [35,44], similar to the “Dynamical Mechanical Excitation” tool described in the literature [24]. Figure 5 describes the schematic of the ESPY33 measurement tool used in this study.

Electric contact probes, which also act as mechanical probes for force loading, are used to measure the piezoelectric charge. In the measurement process, a DC static preload is applied to the ZnO sample while an AC force is administered. The developed charge and force are then recorded through the aixACCT TFA controller to which the ESPY33 tool connects. Full raw charge/current and force data are recorded by the aixACCT system aixacct.com, (accessed on 25 March 2024), and the piezoelectric charge coefficient is calculated, displayed, and recorded. Using Equation (1), the effective piezoelectric coefficient is calculated [24].

(1) $$d_{33}=(\frac{Q}{A_{1}})/(\frac{F}{A_{2}})$$

where *Q* is the piezoelectric charge in Coulombs (C), *F* is the applied force in Newtons (N), and *A*_1_ and *A*_2_ are the electrode and force areas, respectively. In our experiments, a preload of 0.1 N at a signal force of 100 mN was applied to the sample in steps of 10 mN to investigate the load dependence of the piezoelectric response. The results were compared to reference single-crystal piezoelectric materials, quartz and lithium niobate, which were measured under the same conditions [7].

## 3. Results and Discussion

ZnO NW arrays were successfully grown in an aqueous solution using both the conventional CBD and newly built TSG methods. The growth process was preceded by Zn sputtering and subsequent annealing to form a ZnO SL. The morphology of the NW arrays in the TSG approach was controlled by optimizing the SL thickness and the growth temperature, as investigated and presented in our recent work [4]. A ZnO SL thickness of ~10 nm, which was achieved with a Zn thin film sputtering time of 10 min and subsequent annealing in air at 600 °C for 60 min, was used to fabricate the ZnO NWs. The results with the introduction of magnetic stirring saw a reduction in NW dimensions from *L* = 27.35 ± 0.31 µm and *D* = 0.57 ± 0.05 µm to *L* = ~12.66 ± 0.24 µm and *D* = 0.32 ± 0.03 µm. The results with the air pump, however, showed much improvement with an increase in NW dimensions (*L* = 51.86 ± 0.82 µm and *D* = 0.69 ± 0.08 µm and aspect ratio of 75) by a factor of approximately 1.9 for NW length in the TSG standard process (without stirring of the precursor solution). This indicates that with the introduction of an air pump, there is a continuous flow of precursor solution onto the substrate surface, and no precursor precipitation occurs; therefore, there is increased and uniform ZnO NWA growth. The NW dimensions, i.e., length (*L*) and diameter (*D*), were measured using scanning electron microscopy with TESCAN MIRA’s SEM (TESCAN ORSAY HOLDINGS, a. s., Czech Republic). Due to the lack of quantitative information provided by the SEM [45], the NW dimensions were measured using the image processing software ImageJ, version 1.53e.

**Figure 6:**
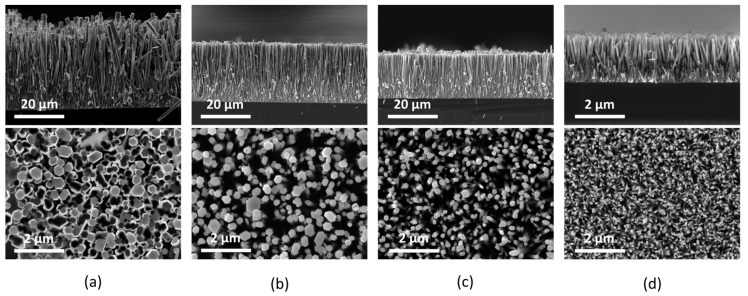
SEM micrographs showing cross-sectional and top views of ZnO NWAs. The ZnO nanowires were synthesized using (**a**) TSG with air pump agitation of precursor solution; (**b**) TSG standard process; (**c**) TSG with magnetic stirring of precursor solution; (**d**) CBD. The growth time for all TSG samples was 24 h, and 3 h for the CBD sample.

The high degree of NW alignment shown in Figure 6 is due to the quality of the ZnO SL resulting from the optimum annealing conditions of the sputtered Zn thin film [6] on the Si substrate surface.

The average length and diameter, as well as the aspect ratio, of the fabricated ZnO NWAs presented in Table 1 were measured from the cross-section and top views of the SEM micrographs in Figure 6. In Table 1, we present the growth conditions and parameters of the ZnO NW samples used in this study.

Using the SEM micrographs from Figure 6, we calculated the density of the NWAs. From a given area for each image, we determined the density of NWs by counting the number of NWs therein and calculating their ratio. The number of NWs was counted using the software CountThings from Photos, version 2.0.8999. As can be expected, the density of NWAs decreases with an increase in NW diameter. This is evident from Table 1, with a NW diameter of ~100 nm having the highest density of 14.6 µm^−2^, and that of ~690 nm diameter being 3.3 µm^−2^.

### 3.1. Indentation Modulus of ZnO Nanowires

Utilizing the AFM system, which is known for its high-resolution capabilities offering detailed insights into the nanoscale behavior of ZnO NWs, the indentation modulus of fabricated NWs was determined for application in piezoelectric energy harvesting. The CR-AFM mode leverages the resonance frequency of the cantilever–NW sample contact to assess mechanical properties such as stiffness and viscoelasticity, which are essential for understanding the electromechanical behavior of the NWs. The following equations were used to calculate the indentation modulus of the fabricated ZnO NWs.

The resonance frequency of a vibrating cantilever in contact with a sample via its tip is related to the contact stiffness k* [29,46,47,48] as follows:

(2) $$k^{*}=2a_{c}E^{*}=\sqrt[{3}]{6E^{*2}RF_{n}}$$

where $a_{c}$ is the contact radius, $F_{n}$ is the normal force on the sample surface, *R* is the radius of the sensor tip, and E* is the reduced Young’s modulus of the tip–sample contact.

(3) $$\frac{1}{E^{*}}=\frac{\left(1-\nu_{S}^{2}\right)}{E_{S}}+\frac{\left(1-\nu_{T}^{2}\right)}{E_{T}}=\frac{1}{M_{S}}+\frac{1}{M_{T}}$$

where $E_{S}$ and $E_{T},$ $\nu_{S}$ and $\nu_{T}$, and $M_{S}$ and $M_{T}$ are the Young’s modulus, Poisson’s ratio, and indentation modulus of the sample and the tip, respectively [29,46,47]. Experimentally, k* can be determined from the calibrated cantilever stiffness, $k_{C}$, and the measured cantilever resonance frequencies in contact with the NW, $f_{NW}$, and free oscillating, $f_{0}$, measured to be 71.77 kHz.

(4) $$k^{*}=k_{C}\left[{\left(\frac{f_{NW}}{f_{0}}\right)}^{2}-1\right]$$

The contact stiffness k* together with Equation (2) yields the reduced Young’s modulus of the tip–NW contact, E*. However, due to the tiny size of the silicon tip, the value of *R* is very difficult to control, since it may continuously change due to the wear or fracture of the tip in contact with the sample. Instead, CR measurements were therefore performed repeatedly at different positions on both the NW sample and a bulk ZnO crystal substrate of known elasticity [49,50]. Based on the mean CR frequencies, $f_{NW}$ and $f_{Ref}$ measured with the ZnO NWA samples and the bulk ZnO reference, respectively, we can calculate corresponding values, $k^{*}$ and $k_{Ref}^{*}$ for the contact stiffness using Equation (4). Rearranging Equation (2), we obtain (with Equation (4)) the reduced Young’s modulus of the tip–NW contact as follows:

(5) $$E^{*}=E_{Ref}^{*}\times \sqrt{\frac{k^{*3}}{k_{Ref}^{*3}}}=E_{Ref}^{*}\times {\left[\frac{{\left(\frac{f_{NW}}{f_{0}}\right)}^{2}-1}{{\left(\frac{f_{Ref}}{f_{0}}\right)}^{2}-1}\right]}^{\frac{3}{2}}$$

where $E_{Ref\;}^{*}$ = 71.1 GPa is the reduced Young’s modulus of the tip–bulk ZnO contact, which was calculated using Equation (3) and the values of 125 GPa and 165 GPa reported for the indentation moduli of bulk (0001) ZnO and the (100) Si tip, respectively [29]. With E* and *M_T,_* we can then calculate the indentation modulus of the ZnO NWs after rearranging Equation (3) according to the following:

(6) $$M_{NW}=\frac{E^{*}{\times M}_{T}}{M_{T}-E^{*}}$$

Using the measured mean values, *f_CR_CBD200_* = 235 ± 2.0 kHz, *f_CR_bulk_* = 245 ± 2.0 kHz (see above), and $E_{Ref\;}^{*}$ = 71.1 GPa, the indentation modulus of the ZnO NWA is calculated using Equations (5) and (6) to be $M_{CBD200}$ = 99.3 ± 2.2 GPa.

**Figure 7:**
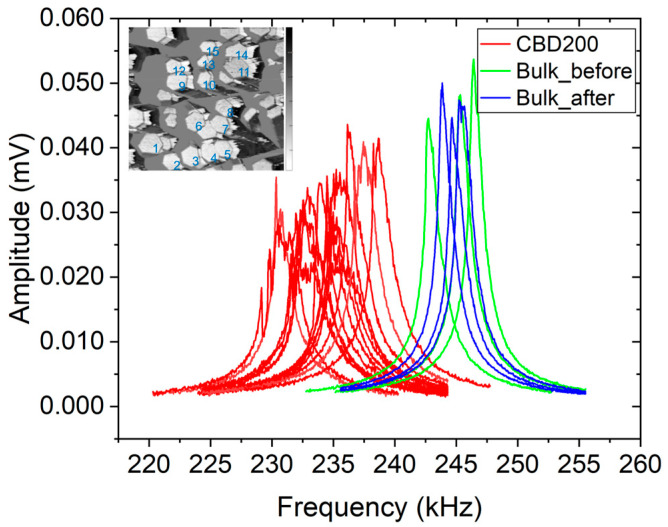
CR measurements of ZnO nanowires (CBD200) with an average diameter of 199 ± 0.07 nm and height of 1.83 ± 0.14 µm, and a bulk ZnO crystal. The inset shows the measured positions on the NWA sample.

The variations in CR frequencies can be attributed to the changing contact area and different diameters of the probed NWs. The dispersed spectrum of NWs, which is visible in Figure 7, extends therefore over a larger frequency range than that of the bulk ZnO reference material. This cannot be attributed to a distinct increase in contact area caused by tip wear, as the CR frequencies measured with the bulk ZnO vary randomly but do show a systematic increase with time. Different pillar heights and the probability of two or more NWs touching one another could also be factors.

**Figure 8:**
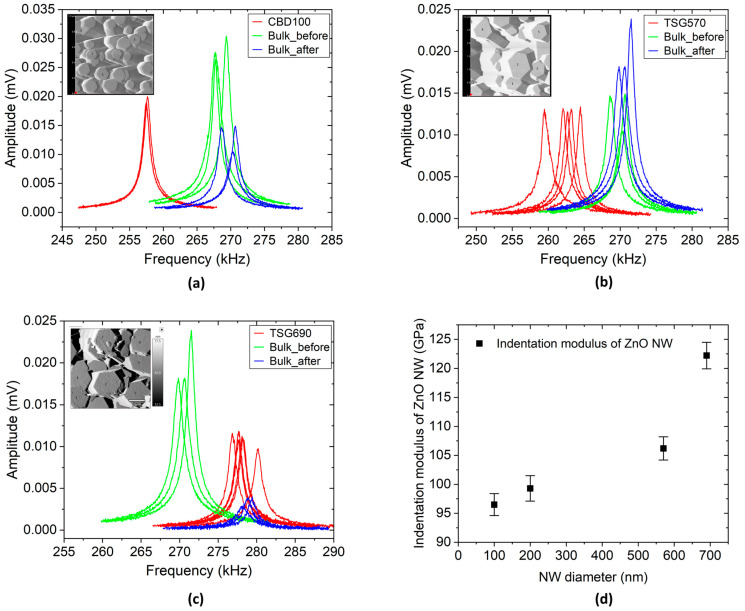
Contact resonance measurement of ZnO nanowires (without embedding polymer) and bulk ZnO crystal substrate: (**a**–**c**) show measured positions (inset) and results of bulk ZnO and ZnO NWAs with diameters 100 nm, 570 nm, and 690 nm, respectively; (**d**) calculated indentation modulus dependence on NW diameter (see Table 2).

Using a resonance frequency *f_CR_CBD100_* = 257.7 ± 2.0 kHz for the CBD100 and *f_CR_bulk_* = 270.3 ± 2.0 kHz for the bulk ZnO (see Figure 8a) and $E_{Ref\;}^{*}$ = 71.1 GPa, the indentation modulus of the ZnO NWA is calculated using Equations (5) and (6) to be $M_{NW100}$ = 96.5 ± 1.9 GPa. In Figure 8b, a resonance frequency, *f_CR_TSG570_* = 262.7 ± 1.7 kHz, was measured for TSG570, and *f_CR_bulk_* = 270.6 ± 1.7 kHz for the bulk ZnO. The calculated indentation modulus of TSG570 is then $M_{TSG570}$ = 106.2 ± 2.0 GPa. It is obvious from Figure 8c that the tip geometry has been distinctly changed before the measurement of NW690 (see Bulk_after vs. Bulk_before). By using *f_CR_TSG690_* = 278.2 ± 1.6 kHz and *f_CR_bulk_* = 279.4 ± 2.7 kHz (in this case we use only the Bulk_after data) the calculation of the indentation modulus of TSG690 yields $M_{TSG690}$ = 122.2 ± 2.3 GPa.

Standard deviations for the indentation moduli, *M*_NW_, were computed by propagating the uncertainties from the resonance frequencies using error propagation. For a function

(7) $$M=f(f_{NW},f_{bulk})$$

while the uncertainty, $\sigma_{M}$, is calculated as follows:

(8) $$\sigma_{M}=\left|\frac{dM}{dE^{*}}\right|\cdot \sigma_{E^{*}}$$

where

(9) $$\sigma_{E^{*}}=E^{*}\cdot \sqrt{{\left(\frac{\sigma_{f_{NW}}}{f_{NW}}\right)}^{2}+{\left(\frac{\sigma_{f_{bulk}}}{f_{bulk}}\right)}^{2}}$$

We can then propagate $\sigma_{E^{*}}$ to $\sigma_{M}$ using

(10) $$\sigma_{M}=\left|\frac{M_{T}^{2}}{{\left(M_{T}-E^{*}\right)}^{2}}\right|\cdot E^{*}\cdot \sqrt{{\left(\frac{\sigma_{f_{NW}}}{f_{NW}}\right)}^{2}+{\left(\frac{\sigma_{f_{bulk}}}{f_{bulk}}\right)}^{2}}$$

Uncertainty values were calculated using error propagation via Equation (10), assuming the errors in frequencies dominate. The tip indentation modulus *M*_T_ = 165 GPa was used, and a reduced modulus for the tip–bulk ZnO contact of E*_Ref_ = 71.1 GPa (calculated using Equation (3) and an indentation modulus of bulk ZnO, $M_{Ref}$ = 125 GPa).

**Table 2:** Results of indentation modulus of ZnO NWs of different lengths and diameters, and bulk ZnO crystal substrate characterized by *CR* measurements (indentation modulus of bulk ZnO, $M_{Ref}$ = 125 GPa [29]) leading to E*_Ref_ = 71.1 GPa using Equation (3) and *M*_T_ = 165 GPa. Uncertainty contributions were computed by error propagation using Equations (9) and (10).

Sample ID	Diameter, *D* (µm)	Length, *L* (µm)	Resonance Frequency, *f_CR_* (kHz)		Reduced Young’s Modulus, E* (GPa)	Indentation Modulus, *M_S_* (GPa)
			ZnO NW	Bulk ZnO		
CBD100	0.10 ± 0.01	2.69 ± 0.05	257.7 ± 2.0	270.3 ± 2.0	60.9	96.5 ± 1.9
CBD200	0.20 ± 0.07	1.83 ± 0.14	235.0 ± 2.0	245.0 ± 2.0	62.0	99.3 ± 2.2
TSG570	0.57 ± 0.05	27.35 ± 0.31	262.7 ± 1.7	270.6 ± 1.7	64.6	106.2 ± 2.0
TSG690	0.69 ± 0.08	51.86 ± 0.82	278.2 ± 1.6	279.4 ± 2.7	70.2	122.2 ± 2.3

The reported values of the Young’s modulus of wurtzite-type ZnO NWs differ drastically between 20 and 800 GPa [51]. Resonant flexural oscillations of ZnO NWs with diameters in the range of 17 to 550 nm and 48–239 nm showed Young’s modulus values larger than the bulk reference value of 140 GPa, with a drastic increase with decreasing diameter [44], which is consistent with the diameter dependence of the radial elastic modulus obtained using CR-AFM [52]. In these cases, NWs were grown using the vapor–solid–liquid (VLS) method, resulting in core–shell morphologies of a bulk-like core and stiffer surface layer(s), which can explain this behavior [35]. Stronger surface atomic bonds in vertical ZnO nanowires grown by the VLS method with diameters ranging from 48 nm to 239 nm lead to an exponential increase in the Young’s modulus towards smaller diameters beyond the bulk reference, as determined from the resonance frequency of the vibrating upstanding NWs [28]. However, ZnO NWs of an average diameter of 45 nm grown by the VLS method investigated as-grown on its substrate using lateral bending in an AFM, showed much smaller Young’s moduli of 29 ± 8 GPa [53]. With ZnO NWs produced by catalyst-free chemical vapor deposition, a core–shell structure was not found from two-point bending vibration spectra, with diameters from 78 to 310 nm. Here, the Young’s modulus varies from 123 to 154 GPa, about the bulk value of 140 GPa, showing no dependence on NW diameter and surface defects [54]. Multiple frequencies of flexural vibrations were observed, and their ratios were compared with the theoretical expectation to check the well clamping and uniform geometry of the measured NWs.

Flexural vibration induces stress at the sidewall surfaces of NWs due to their distance from the neutral axis, i.e., surface elasticity is expected to have a more pronounced effect on the measured effective stiffness than an axial monotonic deformation, as in the case of indentation loading or CR-AFM with upstanding NWs. Correspondingly, lithographically patterned single-crystalline GaN micropillars (*D* ≈ 1.4 µm) investigated using nanoindentation (corrected for the body stiffness of the pillars) and CR-AFM showed elastic moduli (7 to 12)% lower than the bulk reference [48]. The same trend was found with single-crystalline vertical silicon micropillars of (100), (110), and (111) orientations and diameters of 1.6 µm, 1.4 µm, and 1.2 µm, respectively [41]. Microprobe CR imaging (CRI) with ZnO nanowire arrays produced by CBD (diameter of 310 ± 63 nm) revealed an indentation modulus of 129 ± 47 GPa, which is close to the value of bulk ZnO [24]. In this case, the tip radius was much larger than the NW diameter, i.e., contact with a single NW probably did not occur, which may explain the large uncertainty. In comparison with these observations, the present study shows a similar behavior and a trend to be softer compared with its bulk counterpart, as the NW diameter and thus the crystal grain size decreases.

In the contact resonance signals, the *Q*-factor (*f*_CR_/Δ*f*) of the bulk ZnO sample and CBD100, TSG570, and TSG690 have no significant differences. These values are calculated from the center frequency (*f*_CR_) and the full width at half maximum (FWHM) taken as the bandwidth (Δ*f*) of the resonance peaks. By using Gaussian fitting and error propagation, for instance, for CBD100 in Figure 8a with *f*_CR_CBD100_ = 257.7 ± 0.1 kHz and Δ*f* = 2.1 ± 0.1 kHz, its *Q*-factor is estimated at 123 ± 6.3. Considering the resonance frequency value of Bulk_before (green line) from the graph in Figure 8a, i.e., *f*_CR_bulk_ = 267.5 ± 0.1 kHz and given Δ*f* = 2.0 ± 0.1 kHz, the *Q*-factor of the bulk ZnO has an estimated value of 134 ± 6.7. This indicates that the viscoelastic properties of the NWAs are only slightly different from the bulk ZnO. A higher *Q*-factor means the material is more efficient for vibration energy harvesting and sensing applications. The differences in *Q*-factor for each measured NW could result from varied damping, which can be attributed to material fatigue, changes in contact mechanics, or possible contamination.

### 3.2. I-V Characterization

**Figure 9:**
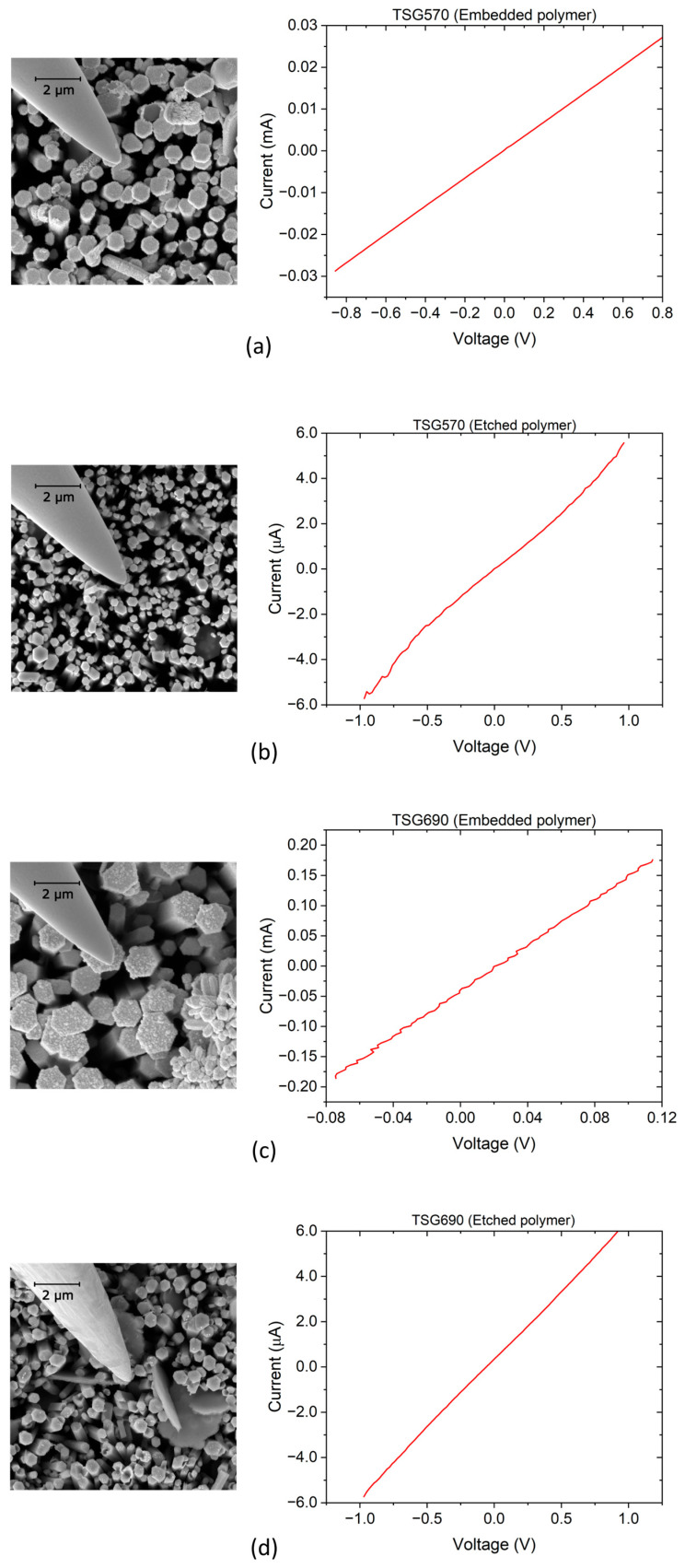
Results of representative *I-V* measurements on ZnO nanowire arrays using a micromanipulator: (**a**,**b**) SEM micrograph of the electrical probe in contact with the NWA sample TSG570 (embedded in polymer matrix and etched polymer) and its corresponding *I-V* graphs, respectively; (**c**,**d**) SEM micrograph of electrical probe in contact with the NWA sample TSG690 (embedded in polymer matrix and etched polymer) and its corresponding *I-V* graphs, respectively. For (**b**,**d**), the embedding polymer was etched in part by O_2_ plasma for contact probing.

Depending on the size of the contact regarding the ZnO nanowires’ cross-sectional width, the electrical behavior of the interface between free-standing ZnO nanowires (semiconductor) and a metal contact on top can change from Schottky to Ohmic [55,56]. The phenomenon has been investigated in this study by using a micromanipulator to measure the *I-V* characteristics of the ZnO NWs in the as-grown state, i.e., without removing them from the substrate. The results of the measured *I-V* characteristics of ZnO NWs with different diameters and types of interfaces formed on the NW top are presented in Figure 9.

From Figure 9a,c,d, the *I-V* curves have a linear characteristic showing an Ohmic top contact by the tungsten probe pressed against the NW top surface. Figure 9b, on the other hand, depicts a wavy *I-V* curve for sample NW690, which can be attributable to an embedding polymer residue after O_2_ plasma etching or a Cr/Au thin film top contact. The effect of the Cr/Au thin film top contact is also evident from the current values, which are much larger than without metallization. Typically, when a tungsten probe directly contacts an *n*-type ZnO NW during electrical probing using a micromanipulator, a nonlinear characteristic is observed, corresponding to a Schottky contact. This is expected due to the difference in the work function of the tungsten probe (~4.6 eV) and the electron affinity of ZnO (~4.3 eV) [27,57]. In this study, three different NWs were investigated for each sample, and three repetitive *I-V* curves were recorded at each NW, which generally exhibits Ohmic behavior. This can be expected if the probe contact radius is smaller than the NW top radius [55].

Nonetheless, several factors may explain the Ohmic contact characteristics depicted in Figure 9. For example, surface states and Fermi level pinning caused by surface defects or absorbates such as oxygen vacancies on ZnO can pin the Fermi level near the conduction band. These could lower the effective Schottky barrier height at the interface and allow Ohmic-like conduction. Furthermore, because the ZnO NW is piezoelectric, mechanical force from the tungsten probe tip can deform the NW during measurement, producing an internal electric field that can change the band structure. This can significantly reduce or even remove the barrier height at the interface, resulting in Ohmic conduction [57]. Another possible explanation is the contamination or alteration of the tungsten tip. An oxide layer, polymer residue, or conductive material on the probe tip may alter the effective work function or contact nature, resulting in Ohmic behavior [57]. Also, the local dielectric breakdown of the embedding polymer under tip bias is likely to create filamentary conduction paths, which mimic short-circuits, leading to linear *I-V* curves [24]. These and other aspects could be linked to the resultant *I-V* curves from the electrical probing measurements in this work, which, without exception, reveal Ohmic properties.

The electrical conductivity of the fabricated ZnO NWs was calculated using the following equation [27]:

(11) $$\sigma =\frac{L}{R\cdot S}$$

where σ is the electrical conductivity, *L* is the length of the nanowire, *R* is the resistance of the nanowire, and *S* is the cross-sectional area of the ZnO nanowire. The value of *S* can be calculated from

(12) $$S=\frac{3}{2}\sqrt{3}a^{2}=\frac{3^{3/2}}{8}D^{2}$$

with a representing the side length of hexagonal ZnO NW given as half the NW diameter *D*. A summary of the electrical conductivity of ZnO NWs that have been measured with micromanipulator-coupled tungsten probes is presented in Table 3.

The assumed uncertainties (in %) for measured NW resistance values are based on the typical characteristics of the measurement setup, which are influenced by contact resistance, tip variability, and instrumentation accuracy. These uncertainties were assumed because of the rather small measurement sample size (*n* = 3), which was not enough to determine the standard deviation. In addition to the known error margins of measured NW dimensions, the uncertainties of the conductivity of NWs were then calculated by error propagation.

With O- and Zn-polar ZnO nanorods produced by CBD in patterned holes in PMMA on O- and Zn-polar ZnO single crystals, electrical conductivity was measured under a flat-lying configuration of the ZnO nanorods via a four-terminal connection using a Ti/Au local Ohmic contact pattern and a multiprobe scanning tunneling microscope (STM), respectively [58]. Zn-polar ZnO NRs are, on average, much more conductive than the O-polar ZnO NRs. In the case of spontaneous NW growth on Zn- and O-polar seeds in the polycrystalline ZnO SL, NWs with both Zn and O polarities can be expected [24]. The conductivity measured with our NWs is mostly in the range of the literature values, except for TSG690 with PMMA as the embedding polymer. In the latter case, short-circuit currents through the polymer may have played a role, which will have a detrimental effect on the piezoelectric output too.

### 3.3. Piezoelectric Coefficient d_33_ of ZnO Nanowires

**Figure 10:**
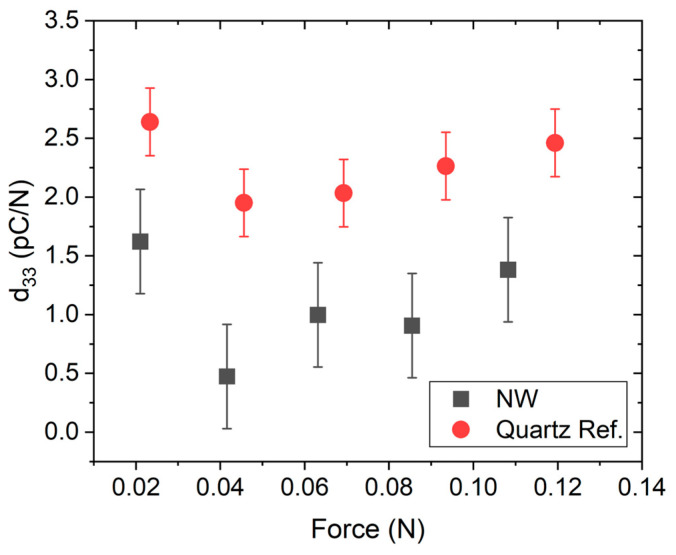
Piezoelectric coefficient of ZnO nanowires (CBD100) measured with an ESPY33 tool.

The piezoelectric and semiconducting properties of a ZnO NW with a diameter of ~100 nm (CBD100) facilitated the piezoelectric *d*_33_ measurement. Maximum/average piezoelectric coefficient values of 1.6 ± 0.4 pC/N and 1.1 ± 0.4 pC/N, respectively, were measured, compared with a value of 3.6 ± 0.5 pC/N reported in our earlier work with an array of NWs with a diameter of ~200 nm [7]. The discrepancy can be associated with the difference in NW diameter and length, with the smaller diameter (but rather longer length) nanowire exhibiting lower *d*_33_ values. Another contributing factor could be the nature of polymer embedding, where, in the previous report, the NWs had a thin layer of SU-8 polymer on top of the NWs. Meanwhile, in this study, the NWs were embedded in S1818 polymer and subsequently etched by O_2_ plasma to reveal the NW tips. Therefore, the mechanical stability in both cases may differ. This assertion is supported by the use of piezoresponse force microscopy (PFM) on a single ZnO NW, where, however, the effective piezoelectric coefficient *d*_33_*^eff^* was observed to have increased with a reduction in NW diameter [18]. Furthermore, PFM characterizes the converse piezoelectric effect instead of the direct piezoelectric effect, i.e., no force is applied on the NWs using PFM. Nevertheless, the current results, compared with our previous result of 3.6 ± 0.5 pC/N for nanowires with a diameter of ~200 nm [7], suggest that the effective piezoelectric *d*_33_ increases with NW diameter, which was not to be expected. Reduced piezoelectric screening due to carrier depletion at the sidewalls caused by surface charges will be more effective for small-diameter NWs [18]. Therefore, the piezoelectric output should increase with the increasing length and decreasing diameter of the NW, which contrasts with our experimental findings. Nevertheless, as discussed in the work of Song et al., a bending strain due to the rather long and too thin NW may have reduced the direct piezoelectric axial response [59]. Again, surface effects tend to dominate at NW diameters from ~100 nm and below, and at a certain point, the piezoelectric response may deteriorate. As the diameter decreases, the effective *d*_33_ can either increase or decrease, depending on surface stress relaxation and the presence of surface defects or adsorbates [60,61]. Furthermore, only embedding the NWs with S1818 (which may not have seeped down and encapsulated the NWs) without a thin polymer layer on top may have caused a short-circuit [24], leading to more resistive instead of capacitive behavior in the case of the present study. Figure 10 shows the results of piezoelectric *d*_33_ for the CBD100 NW sample that was measured in this study.

In Table 4 below, a comparison of some measured piezoelectric *d*_33_ values of ZnO NWs reported in the literature is presented.

## 4. Conclusions and Outlook

ZnO nanowire fabrication using both conventional CBD and newly developed TSG methods was employed in this investigation. Both processes were preceded by Zn metal sputtering and subsequent annealing in air to form ZnO SL. A SL thickness of ~20 nm after 20 min sputtering was realized for the CBD method, while for the TSG method, sputtering was performed for 10 min, resulting in a ~10 nm thick SL. The CBD process was performed at 90 °C for 3 h, while the TSG method took 24 h at 105 °C. SEM micrographs of ZnO NWs from each method show high-density and vertically aligned nanowires, with the TSG method providing longer nanowire dimensions up to ~ 52 µm and an aspect ratio of 75.

The fabricated ZnO NWs were investigated for their elastic modulus using an AFM operated in contact resonance mode. The calculated results indicate that the indentation modulus of individual NWs is dependent on the NW size, with larger diameter NWs (*D* = ~690 nm) recording higher values of up to 122.2 ± 2.3 GPa, close to the reference bulk ZnO value of 125 GPa. This is an indication that the fabricated NWs are comparable and of high quality.

*I-V* measurements of the nanowires with an electrical probing setup show linear characteristics representing Ohmic behavior when the tungsten (W) probe touches the top surface of the nanowire. Similar behavior was observed for both NWAs completely embedded in a polymer matrix and with polymer embedding etched by O_2_ plasma to reveal the NW tips. This is an indication that the embedding polymer, which serves to provide mechanical stability to the nanowire arrays and to avoid short-circuiting, did not have a significant influence on the electrical behavior of the nanowire as long as the top conductive electrode was in contact with the nanowire top surface. However, the realization of Ohmic contact behavior in these measurement results needs further investigation to understand the dynamics in the metal–semiconductor interface. As it is typically expected, a tungsten tip contact to ZnO NW shows supposedly Schottky behavior due to the high difference in work function between the tungsten probe (~4.6 eV) and the electron affinity of ZnO (~4.3 eV) [27,57]. In any case, possible explanations enumerated for a generated electric field by the tungsten tip, and contamination by an oxide layer or conductive material, should not be overlooked.

In a further electrical characterization, we measured the piezoelectric coefficient *d*_33_ of CBD-produced NWs with an average diameter of 100 nm, yielding a maximum value of 1.6 ± 0.4 pC/N and an average of 1.1 ± 0.4 pC/N. The results roughly agree with the reported values of 3.6 pC/N and ~ 1.2 pC/N to ~3.5 pC/N for NW diameters of ~200 nm [7] and ~85 nm [24], respectively, indicating the accurate, consistent, and reliable measurement capability of the traceable ESPY33 tool. The single result (CBD100) on piezoelectric characterization is shown here as proof of a successful device technology and the validity of the ESPY33 tool. Nevertheless, the expected increase in piezoelectric *d*_33_ with increasing NW length and decreasing diameter, which shows the contrary in this study, requires further investigation. Then, the aforementioned factors, including surface defects, bending strain, and influences of the embedding polymer, must not be overlooked. Fabrication and *d*_33_ testing, using the d33 ESPY33 tool, of a series of VINGs of different dimensions and embedding/covering polymers will be shown in our next study. In a future study, we will further investigate the piezoelectric response of ZnO NWs fabricated using high-temperature treatment in the range of (600–900) °C [26].

## Figures and Tables

**Table 1:** Parameters of ZnO NWAs synthesized under different conditions that were used in the investigation.

Sample ID	Sputtering Time	ZnO SL (~nm)	Growth Method	Length—*L* (µm)	Diameter—*D* (µm)	Aspect Ratio	Density (µm^−2^)	Vertical Alignment (°)	Remarks
CBD100	20 min in Ar	20	CBD: 90 °C; 3 h	2.69 ± 0.05	0.10 ± 0.01	27	14.6 ± 10.6	89.8 ± 0.3	Magnetic stirring
CBD200	20 min in Ar	20	CBD: 90 °C; 3 h	1.83 ± 0.14	0.20 ± 0.07	8	10.1 ± 0.8	90.0 ± 0.9	Magnetic stirring
TSG320	10 min in Ar	10	TSG: 105 °C; 24 h	12.66 ± 0.24	0.32 ± 0.03	39	4.0 ± 1.0	89.6 ± 0.4	Magnetic stirring
TSG570	10 min in Ar	10	TSG: 105 °C; 24 h	27.35 ± 0.31	0.57 ± 0.05	48	3.8 ± 5.8	89.7 ± 0.3	Still solution
TSG690	10 min in Ar	10	TSG: 105 °C; 24 h	51.86 ± 0.82	0.69 ± 0.08	75	3.3 ± 2.1	89.5 ± 0.9	Air pump

**Table 3:** Electrical conductivity of ZnO NWs measured with micromanipulators integrated with tungsten (W) probes.

NW Diameter, *D* (nm)	NW Length, *L* (µm)	Polymer/Metallization/Probe	NW Resistance, *R* (kΩ)	NW Conductivity, *σ* (kS/m)	Ref.
690 ± 0.08	52 ± 0.8	PMMA/Cr/Au/W	3 ± 3%	57.1 ± 2.10	TSG690
570 ± 0.05	27 ± 0.3	PMMA/Cr/Au/W	36 ± 2.5%	2.22 ± 0.07	TSG570
570 ± 0.05	27 ± 0.3	etched PMMA/no/W	217 ± 0.5%	0.37 ± 0.004	TSG570
690 ± 0.08	52 ± 0.8	etched PMMA/no/W	202 ± 0.5%	0.83 ± 0.014	TSG690
1060	31 ± 3	no/no/W	-	0.4809	[27] *
420–640	3.963 ± 0.060	no/TiAu/W, Zn-polar	-	5–25	CBD, [58]
		no/no/W, Zn-polar	-	~17	
440–600	3.242 ± 0.085	no/TiAu/W, O-polar	-	0.2–14.3	
		no/no/W, O-polar	-	6–20	

* Commercial source of ZnO nanorods.

**Table 4:** Comparison of measured piezoelectric *d*_33_ values of ZnO nanowires.

NW Diameter, *D* (nm)	NW Length, *L* (µm)	Polymer	*d*_33_ (pC/N)	Ref.
100 ± 10	2.69 ± 0.05	S1818, embedding the NWs, etched by 0.27 µm to remove it from the top	1.6 ± 0.4, max.	CBD100
			1.1 ± 0.4, ave.	
200 ± 70	1.83 ± 0.14	SU-8, ~0.1 µm on top of NWA	1.9 ± 0.5	[7]
			2.1 ± 0.5	
			3.6 ± 0.5	
84 ± 16	1.031 ± 0.016	PMMA, 1.5 µm on top of NWA	3.53 ± 0.33	[24]
		PMMA, 2 µm on top of NWA	1.21 ± 0.16	

## Data Availability

The data presented in this study is available on request from the corresponding author.

## Abbreviations

The following abbreviations are used in this manuscript:

ZnO	Zinc oxide
NWA	Nanowire array
SEM	Scanning electron microscopy
CR	Contact resonance
AFM	Atomic force microscopy
I-V	Current–voltage
PENG	Piezoelectric nanogenerator
TSG	Thermo-convective solution growth
CBD	Chemical bath deposition
SL	Seed layer
DC	Direct current
MEMS	Microelectromechanical systems
SAG	Selective area growth
XRD	X-ray diffraction
VING	Vertically integrated nanogenerator
ICP	Inductive coupled plasma
EBIC	Electron beam-induced current
APT	Advanced probing tool
VLS	Vapor–liquid–solid
CRI	Contact resonance imaging
FWHM	Full width at half maximum
STM	Scanning tunneling microscope
